# The Antiproliferative and Apoptosis-Inducing Effects of the Red Macroalgae *Gelidium latifolium* Extract against Melanoma Cells

**DOI:** 10.3390/molecules26216568

**Published:** 2021-10-30

**Authors:** Eka Sunarwidhi Prasedya, Nur Ardiana, Hasriaton Padmi, Bq Tri Khairina Ilhami, Ni Wayan Riyani Martyasari, Anggit Listyacahyani Sunarwidhi, Aluh Nikmatullah, Sri Widyastuti, Haji Sunarpi, Andri Frediansyah

**Affiliations:** 1 Bioscience and Biotechnology Research Centre, Faculty of Mathematics and Natural Sciences, Mataram University, Mataram 83126, Indonesia; ekasprasedya@unram.ac.id (E.S.P.); nanaardiana36@gmail.com (N.A.); padmi0116@gmail.com (H.P.); baiqtrikhairinailhami@ymail.com (B.T.K.I.); riyani.martyasari@gmail.com (N.W.R.M.); eka.ugm@gmail.com (H.S.); 2 Department of Pharmacy, Medical Faculty, University of Mataram, Mataram 83126, Indonesia; anggit.sunarwidhi@unram.ac.id; 3 Faculty of Agriculture, University of Mataram, Mataram 83125, Indonesia; aluh_nikmatullah@unram.ac.id; 4 Faculty of Food Science and Agroindustry, University of Mataram, Mataram 83125, Indonesia; sriwidyastuti@unram.ac.id; 5 Pharmaceutical Institute, Eberhard Karls University of Tuebingen, 72074 Tuebingen, Germany; 6 Research Division for Natural Product Technology (BPTBA), Indonesian Institute of Sciences (LIPI), Wonosari 55861, Indonesia; 7 National Research and Innovation Agency (BRIN), Wonosari 55861, Indonesia

**Keywords:** brassicolene, B16-F10, cytotoxicity, *Gelidium latifolium*, macroalgae

## Abstract

The red macroalga *Gelidium latifolium* is widely distributed in the coastal areas of Indonesia. However, current knowledge on its potential biological activities is still limited. In this study, we investigated the potential bioactive compounds in *Gelidium latifolium* ethanol extract (GLE), and its cytotoxic effects against the murine B16-F10 melanoma cell line. GLE shows high total phenolic content (107.06 ± 17.42 mg GAE/g) and total flavonoid content (151.77 ± 3.45 mg QE/g), which potentially contribute to its potential antioxidant activity (DPPH = 650.42 ± 2.01 µg/mL; ABTS = 557.01 ± 1.94 µg/mL). ESI-HR-TOF-MS analysis revealed large absorption in the [M-H]^-^ of 327.2339 m/z, corresponding to the monoisotopic molecular mass of brassicolene. The presence of this compound potentially contributes to GLE’s cytotoxic activity (IC_50_ = 84.29 ± 1.93 µg/mL). Furthermore, GLE significantly increased the number of apoptotic cells (66.83 ± 3.06%) compared to controls (18.83 ± 3.76%). Apoptosis was also confirmed by changes in the expression levels of apoptosis-related genes (i.e., *p53*, *Bax*, *Bak*, and *Bcl2*). Downregulated expression of *Bcl2* indicates an intrinsic apoptotic pathway. Current results suggest that components of *Gelidium latifolium* should be further investigated as possible sources of novel antitumor drugs.

## 1. Introduction

Malignant melanoma is the most aggressive form of skin cancer, and currently accounts for approximately 3% of all cases of malignant tumors [1]. The most common carcinogen responsible for the development of melanoma is ultraviolet radiation (UVR) [2]. The global incidence of melanoma is increasing due to the increase in UVR reaching the Earth’s surface [3]. Currently, melanoma, as with other types of cancer, is mainly treated with radiation or chemotherapy. The main problems with these treatments include severe adverse effects and the development of multidrug resistance [4]. Hence, various phytochemicals obtained from natural resources have been extensively investigated for their anticancer activities due to their safety and low-to-moderate toxicity compared to conventional cancer chemotherapeutic agents [5].

Marine macroalgae, also referred to as seaweeds, are well known as a rich source of structurally diverse bioactive compounds with various biological activities, as well as for their importance as a source of novel bioactive substances [6,7,8]. Several studies have revealed that seaweeds have biological activity against cancer, cardiovascular-related diseases, diabetes, inflammation, thrombosis, and obesity [9,10,11,12,13,14,15,16]. In particular, red macroalgae are a critical source of numerous bioactive compounds—unlike other groups of green and brown macroalgae. Bioactive compounds that are commonly present in red macroalgae—such as sulfated galactans, carrageenans, agars, phytoalexins, unsaturated fatty acids, terpenoids, and amino acids—have demonstrated anticancer activity in some cancer cell models [9,17,18,19,20,21,22].

Several other Gelidiaceae family members have been reported to have a variety of biological activities, including the prevention of cancer and cardiovascular disease, lowering blood pressure, and lowering blood glucose levels [23,24,25,26,27]. The red macroalgae genus *Gelidium* is mainly known as the source of agars that are used in the food industry. Approximately 20% of the largest agar industrial sources come from *Gelidium* spp. The agar content of *Gelidium amansii* has demonstrated anti-obesity, antioxidant, and anticarcinogenic activity. However, information regarding other phytochemical constituents in *Gelidium* species remains largely unknown. The species *Gelidium latifolium* is one of the most abundant red macroalgae in coastal areas of West Lombok, Indonesia; however, there remains limited information regarding its potential biological activities. In this study, we evaluated the phytochemical constituents of *Gelidium latifolium* ethanol extract (GLE) and its antiproliferative effect against melanoma cells using murine B16-F10 cells as a melanoma cell model.

## 2. Materials and Methods

### 2.1. Macroalgal Material

The red macroalga *Gelidium latifolium* was collected from the Batu Layar coast, West Lombok, Indonesia (8°24’11.7396” S, 116°4’1.9056” E). The macroalgae samples were identified based on an electronic algae database [28]. Corresponding material was deposited in the herbarium of Pusat Unggulan Biosains dan Bioteknologi (PUBB), University of Mataram. Fresh samples were washed with distilled water to remove adhering debris. Cleaned samples were then sprayed with 1% fungicide (Rely+On, Virkon) to prevent fungal contamination. The macroalgae samples were then dried at room temperature (24 °C) with air conditioning. After approximately 5 days, the macroalgae samples were incubated in an oven (Binder ED 56) at 40 °C until reaching a constant weight [29]. Dried samples were then ground with a blade grain miller (food-grade stainless steel) and kept in powder form at 4 °C until further use [30].

### 2.2. Chemicals and Reagents

For extraction, ACS-grade 96% ethanol (Merck, Germany) was used. The MTT (3-(3,5-dimethylthiazol-2-yl)2,5-diphenyltetrazolium) reagent was purchased from BioVision, Inc. (Milpitas, CA, USA). Dulbecco’s modified Eagle medium (DMEM), fetal bovine serum (FBS) (South American origin), and penicillin were purchased from Gibco, Life Technologies (Paisley, UK). The fluorescence probes calcein-AM, propidium iodide (PI), and Hoechst33342 were purchased from Dojindo, Japan.

### 2.3. Preparation of Gelidium Latifolium Ethanol Extracts

Fifty grams (50 g) of dried algal powder was mixed with absolute ethanol solvent at a volume of five times the volume of the sample weight (*w*/*v*). The algal sample was extracted by maceration and incubated at room temperature for 3 × 24 h with constant stirring. After every 24 h, the mixture was filtered with Whatman No. 1 filter paper. All of the filtrates were collected and evaporated with a rotary evaporator (40 °C, 50 rpm) to evaporate the ethanol solvent. The resulting pasty extracts were collected and stored at 4 °C until future use [31].

### 2.4. Determination of GLE’s Total Phenolic Content and Total Flavonoid Content

The total phenolic content (TPC) of GLE was determined via the Folin–Ciocalteu colorimetric method [32]. Gallic acid equivalents (GAE) were used as the standard. A stock solution of GAE was prepared by dissolving 10 mg with 10 mL of ethanol (1 mg/mL). A dilution series of GAE concentrations (10–500 µg/mL) was prepared for the generation of the standard curve. An exact amount of 100 µL of sample (1 mg/mL) was combined with 0.75 mL of the Folin–Ciocalteu reagent (diluted 10-fold in distilled water before use). The mixture was incubated at room temperature for 5 min. A volume of 750 µL of sodium carbonate (Na_2_CO_3_) was added to the mixture and mixed gently with pipetting. The reaction took ~90 min. Absorbance was measured at 725 nm with a UV–Vis spectrophotometer microplate reader (Multiskan GO, Thermo Fisher Scientific). The TPC value obtained was revealed as gallic acid equivalents per 100 g of the dry extract.

The total flavonoid content (TFC) was measured according to the method described by Aregan et al., with minor modifications [33]. A volume of 100 µL of sample was added, along with 4 mL of distilled water, followed by the addition of 300 µL of 5% sodium nitrite. After 5 min, 300 µL of 10% aluminum chloride was added. The mixture was incubated for an additional 6 min before the addition of 2 mL of 1 M sodium hydroxide. Immediately, the mixture was diluted by the addition of 3.3 mL of distilled water, and then vortexed. The absorbance was determined at 510 nm versus a blank. Quercetin was used as the standard for the calibration curve. The total flavonoid content of the sample was expressed as mg of quercetin equivalents per 100 g of the dry extract.

### 2.5. Antioxidant Activity of GLE (DPPH and ABTS Assays)

The determination of GLE antioxidant capacity was based on DPPH (2,2-diphenyl-2-picrylhydrazyl) radical scavenging activity [34]. A volume of 100 µL of GLE or ascorbic acid (AA) at various concentrations (10–4000 µg/mL) was mixed with 100 µL of 200 µM freshly prepared DPPH in methanol. The reaction was conducted in the dark at room temperature for 30 min, and then the absorbance was measured at 517 nm. The measurement was repeated three times, and free-radical-inhibiting activity was calculated by Equation (1):

(1) $$Scavenging\;activity\;\left(\%\right)=\left(\frac{A_{517}\;of\;control-A_{517}\;of\;sample}{A_{517}\;of\;control}\right)\times 100$$

The ABTS (2,2’-azinobis(3-ethylbenzothiazolin-6-sulfonic acid)) radical cation method was also used to determine the antioxidant activity of GLE. The ABTS reagent was prepared by mixing 5 mL of 7 mM ABTS with 88 µL of 140 mM potassium persulfate [35]. The mixture was incubated in the dark at room temperature for 24 h to allow the generation of free radicals. For measurement of the scavenging activity of macroalgae extracts based on ABTS assay, a volume of 100 µL of ABTS reagent was mixed with 100 µL of sample or ascorbic acid (standard) in a 96-well microplate and incubated at room temperature for 6 min. After incubation, the absorbance was measured at 734 nm using a UV–Vis spectrometer (Multiskan GO, Thermo Fisher Scientific). The experiment was repeated three times, and the ABTS scavenging activity was measured using Equation (2):

(2) $$Scavenging\;activity\;\left(\%\right)=\left(\frac{A_{734}\;of\;control-A_{734}\;of\;sample}{A_{734}\;of\;control}\right)\times 100$$

### 2.6. Electrospray Ionization High-Resolution Time-of-Flight Mass Spectrometry (ESI-HR-TOF-MS)

High-resolution mass spectra were provided by a Bruker Daltonics maXis 4G time-of-flight mass spectrometer with electrospray ionization (ESI-HR-TOF-MS). Approximately 5 µL of GLE was subjected to the reversed phase of a C18 column (Phenomenex^®^ Luna Omega 1.6 µm Polar C18, 100 Å, 150 *×* 2.1 mm) with a flow rate of 0.75 mL/min to disperse the analytes. Mass spectra (MS) analysis was performed in negative mode (50 eV). MS-grade 90% water with 0.1% formic acid to 100% MS-grade acetonitrile with 0.06% formic acid was used.

### 2.7. B16-F10 Murine Melanoma Cell Culture

B16-F10 murine melanoma cells (ECACC 92101204) and NIH-3T3 normal murine fibroblasts (ECACC 93061524) were purchased from the European Collection of Authenticated Cell Cultures. The cells were routinely cultivated in Dulbecco’s modified Eagle medium (DMEM, Wako) supplemented with 10% FBS for B16-F10 and 5% for NIH-3T3. The cells were kept at 37 °C in a 5% CO_2_ humidified incubator (Forma Steri-Cycle, Thermo Fisher Scientific). For all experiments, cells were grown in T-25 cell culture flasks with a seeding density of 0.8 × 10^6^ cells/mL. After reaching 80–90% confluence, cells were then seeded according to the experimental requirements.

### 2.8. Cytotoxicity Assay

Cytotoxicity was estimated via the MTT cytotoxic assay [36]. B16-F10 and NIH-3T3 cells were cultured in 96-well culture plates at a seeding density of 1 × 10^4^ cells/well. After 24 h, the culture media were discarded and replaced with new media containing various concentrations of GLE or doxorubicin (1–1000 µg/mL). After 72 h of incubation, the wells were supplemented with 50 µL of MTT reagent in 50 µL of serum-free DMEM. The 96-well plates were then kept at 37 °C with 5% CO_2_ for 3 h. After 3 h of incubation, the solution was discarded and replaced with 150 µL of MTT solvent, and then rotated for 15 min. Color change depending on MTT activity was then measured by absorbance at 590 nm with a UV–Vis microplate reader (Multiskan GO, Thermo Fisher Scientific). Cytotoxicity was calculated by Equation (3). The absorbance of the control group at 590 nm (A_590 control_) refers to the absorbance of the wells with no GLE or doxorubicin. A_590 treated cells_ refers to the absorbance of the wells treated with various concentrations of GLE or doxorubicin. All experiments were performed in triplicate. Cell morphology was observed at 20× magnification with a phase-inverted microscope (Zeiss primo vert, Zeiss, Germany).

(3) $$Cytotoxicity\;\left(\%\right)=\left(\frac{A_{590}\;treated\;cells}{A_{590}\;control}\right)\times 100$$

### 2.9. Calcein-AM and Propidium Iodide Staining (Viability Analyses)

Calcein acetoxymethyl (calcein-AM) and propidium iodide (PI) viability staining were performed to determine the cells’ apoptotic features [9]. The cells were seeded at a density of 3 × 10^4^ cells/well in a 35 mm dish at 37 °C and a 5% CO_2_ atmosphere in 2 mL media (DMEM supplemented with 10% FBS and 1% penicillin). After 24 h of incubation, the medium was replaced with medium containing GLE or doxorubicin at IC_50_ concentration. After 72 h, the cells were washed twice with PBS, followed by the addition of calcein-AM (5 µM) and PI (5 µM). The stained cells were incubated for 15 min at 37 °C before observation with a fluorescence-inverted microscope (Axio observer Z1, Zeiss, Germany). Cells that emitted green fluorescence were live cells, whereas the cells that emitted red fluorescence were dead cells [37]. Further cell analysis was conducted using ImageJ software.

### 2.10. GLE’s Effect on DNA Condensation (Hoechst33342 Nuclear Staining)

Staining cells with the fluorescence dye Hoechst33342 is one of the ideal assays to determine apoptotic events in cells [38]. The B16-F10 cells were grown in 35 mm tissue culture dishes at a seeding density of 0.3 × 10^6^ cells/mL. After 24 h, the media were discarded and replaced with non-treated medium or media containing GLE IC_50_ or doxorubicin IC_50_. The cells were then incubated for another 72 h at 37 °C with 5% CO_2_. After 72 h, the media were discarded and replaced with culture medium containing 40 µM of Hoechst33342. The cells were then incubated for 20 min at 37 °C with 5% CO_2_. Finally, the cells were washed twice with PBS and observed under a fluorescence-inverted microscope with 350 nm excitation filters. The cell images were taken at 20× magnification and analyzed with ImageJ to determine the apoptotic nuclei percentage. The fluorescence intensity of condensed nuclear DNA was determined using the corrected total cell fluorescence (CTCF) equation [39]: CTCF = integrated density—(area of selected cell × mean fluorescence of background readings).

### 2.11. GLE’s Effect on DNA Fragmentation

One of the biochemical hallmarks of apoptosis is the condensation and fragmentation of genomic DNA [40]. This is an irreversible event that commits the cell to die, and occurs before changes in plasma membrane permeability. The cells were seeded in 24-well plates for 24 h at a seeding density of 5 × 10^4^ cells/mL. The next day, the culture medium was replaced with an IC_50_ concentration of GLE or doxorubicin and incubated for a further 72 h. After 72 h of incubation, the total cellular DNA was extracted with the DNeasy Kit (Qiagen, USA). Retrieved genomic DNA was run in 1.5% agarose and subjected to electrophoresis (80 V, 40 min). A DNA size marker of 1 kb (GeneRuler 1 kb, Thermo Scientific) was used as the standard DNA ladder. The gels were documented with the GelDoc imaging system (Cambridge, UK).

### 2.12. RNA Isolation and Semi-Quantitative PCR Analysis

Total RNA was extracted from both untreated and treated B16-F10 cells using the RNeasy Mini Kit (Qiagen, Valencia, CA, USA). The B16-F10 cells were seeded in 24-well plates at a cell density of 5 × 10^4^ cells/mL. After 24 h of incubation, the medium was replaced with an IC_50_ concentration of GLE or doxorubicin and incubated for a further 72 h. The total RNA was isolated from B16-F10 cells according to the manufacturer’s instructions. The obtained RNA was then converted to cDNA with a PrimeScript 1st strand cDNA synthesis kit (Takara, Japan), and PCR was performed using a TopTaq Master Mix PCR kit (Qiagen, USA). The expression of the apoptosis-related genes *p53*, *bak*, *bax*, and *Bcl2* was investigated using primers that corresponded to data in GenBank. The primers were ordered from Fasmac, Japan. The semi-quantitative analyses of PCR products were determined relative to the housekeeping gene (*GAPDH*) with Image Lab software (Bio-Rad, Hercules, CA, USA).

### 2.13. Statistical Analyses

One-way ANOVA followed by Tukey’s multiple comparison post hoc test was conducted for multiple comparisons between treatment groups and controls. Experiments were repeated at least three times, and the data were represented as the mean ± SD. All statistical analyses were performed using GraphPad Prism software ver.9.2.0 (San Diego, CA, USA). A *p*-value of less than 0.05 was considered to be statistically significant, whereas a *p*-value of less than 0.01 was considered to be highly significant.

## 3. Results

### 3.1. The Morphology and Phytochemical Analysis of GLE

**Figure 1:**
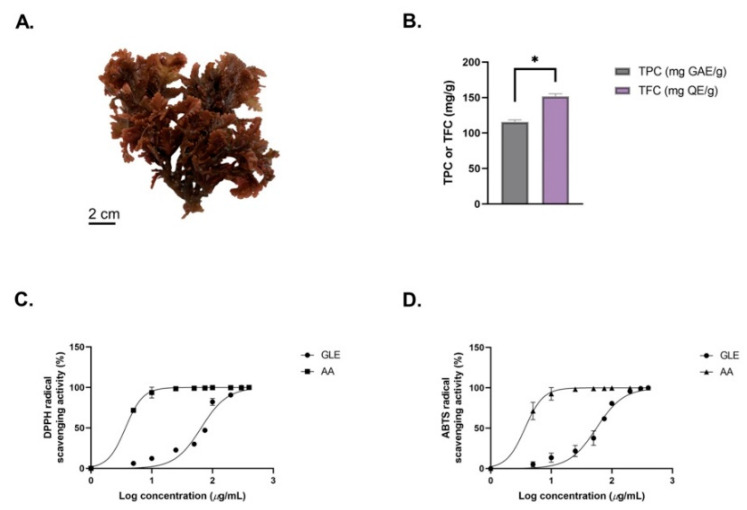
(**A**) Morphological features of the red seaweed *Gelidium latifolium*. (**B**) Total phenolic content (TPC) and total flavonoid content (TFC) of GLE. (**C**) DPPH radical scavenging activity of GLE. (**D**) ABTS radical scavenging activity of GLE. Data represented are the mean ± SEM of 3 independent experiments; * indicates significant differences between groups (*p* < 0.05).

The macroalga *Gelidium latifolium* has a dark-red-colored thallus with a relatively small morphological size ranging from 5 to 7 cm (Figure 1A). The extract yield obtained from extraction of 50 g of dried *G. latifolium* powder was 0.87 ± 0.15 g. Using the Folin–Ciocalteu method, the total phenolic content (TPC) of *G. latifolium* was investigated. In this study, the red alga *G. latifolium* was found to obtain TPC of 107.06 ± 17.42 mg GAE/g (Figure 1B). Previous research has found lower TPC levels in ethanolic extracts of *Sargassum muticum* (94.20 mg GAE/g), *Turbinaria conoides* (0.09 mg GAE/g), *Laminaria ochroleuca* (83 mg GAE/g), and *Halopteris scoparia* (2.92 mg GAE/g) [32,33]. Other findings in relation to *Gelidium* species also reported low TPC in *Gelidium* sp. (6.33 mg GAE/g) and *Gelidium chilense* (9.9 ± 1.3 mg GAE/g) [34,41]. These differences could be influenced by numerous factors, such as species, season, age, environmental conditions, and extraction methods [42]. This could be seen in the study by Alvarez-Gomez et al., where the red alga *Gelidium pusillum* was reported to contain a higher amount of phenolic compounds compared to the related species *Gelidium corneum* [43].

The TFC of GLE was also determined, using quercetin (QE) as standard (Figure 1B). The TFC value of GLE (151.77 ± 3.45 mg QE/g) was higher compared to *Sargassum vulgar* (187 ± 5 mg QE/g) and *Gracilaria corticata* (105 ± 10 mg QE/g) [44]. In addition, GLE showed significantly higher TFC compared to ethanolic extract of *Gelidium pusillum* (4.46 ± 0.004 mg QE/g) [45]. GLE’s potential antioxidant activity was determined based on the DPPH and ABTS assays (Figure 1C). The antioxidant IC_50_ values (DPPH = 650.42 ± 2.01 µg/mL; ABTS = 557.01 ± 1.94 µg/mL) obtained from the ethanolic crude extract of *G. latifolium* were lower compared to previous findings. A previous study by Chakraborty et al. reported that the methanolic extracts of *H. musciformis*, *H. valentiae*, and *J. rubens* had TFCs above 2 mg/mL [46]. The antioxidant activity IC_50_ value of GLE was also lower compared to crude extracts of other wild red macroalgae, such as *Pterocladiella capillacea* (7.90 ± 0.04 mg/mL), *Hypnea spinella* (11.59 ± 0.05 mg/mL)*, Dermocorynus dichotomus* (4.69 ± 0.01 mg/mL), *Halopithys incurva* (2.62 ± 0.02 mg/mL)*,* and *Laurencia dendroidea* (2.48 ± 0.01 mg/mL) [47]. Meanwhile, the IC_50_ value of 200–1000 µg/mL from crude extracts could be considered to have potential antioxidant activity [48]. Hence, this highlights the antioxidant potential of *G. latifolium*, which correlates with its high phenolic and flavonoid contents.

### 3.2. Brassicolene Detected in GLE by ESI-HR-TOF-MS

**Figure 2:**
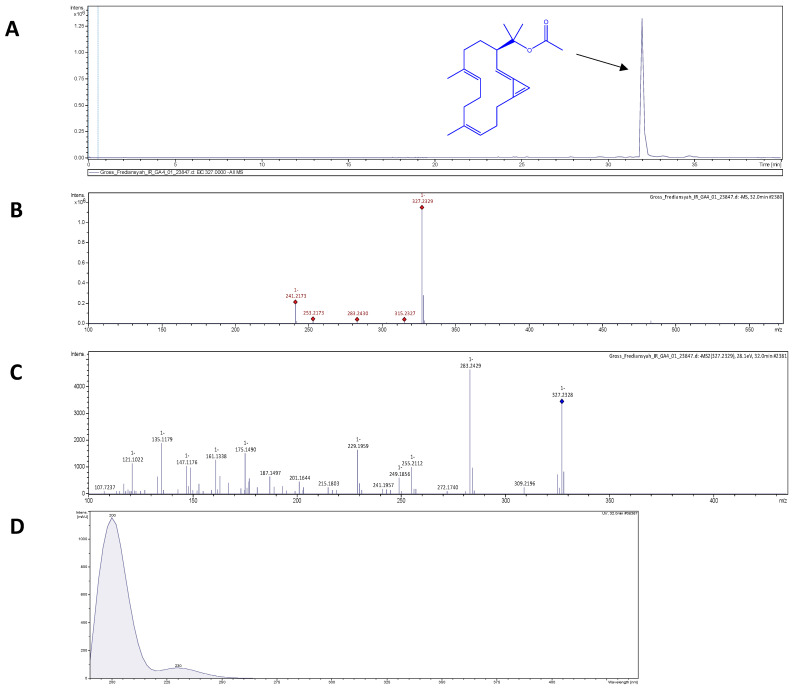
ESI-HR-TOF-MS analysis of putative brassicolene in GLE: (**A**) Extracted ion chromatogram and chemical structure of brassicolene (a m/z of 327.2339 [M-H]^-^). (**B**) Negative mode MS^1^ of brassicolene. (**C**) Negative mode MS^2^ of brassicolene. (**D**) UV absorption in mass spectrometric analysis of brassicolene.

ESI-HR-TOF-MS was used to assess the phytochemical composition of GLE. GLE extract contains putative bioactive compounds including 2H-pyran; 5-methyl-2-octyl-3(2H)-furanone; trans, cis-2,6-nonadienal; 12-oxo-trans-10-dodecenoic acid; palmitoleic acid, (2E)-2-tetradecenal; and eicosenoic acid (Figure S1). Therefore, the main component was probably brassicolene, or a closely related molecule with [M-H]^−^ of 327.2339 m/z—as shown in Figure 2A–C, which depicted (A) the extracted ion chromatogram of putative brassicolene, and (B,C) the MS^1^ and MS^2^ data, respectively. Brassicolene has the chemical formula C_22_H_32_O_2_, with a mass error of 0.2 ppm, and a round double bond (rdb) of 7, signifying seven degrees of unsaturation. Furthermore, UV absorption at 200 nm confirmed the presence of a conjugated diene (Figure 2D). The ESI-HR-TOF-MS and UV analyses of putative brassicolene found in GLE were consistent with the findings of Duh et al. [49].

### 3.3. Cytotoxic Activity of GLE on B16-F10 Melanoma Cells

**Figure 3:**
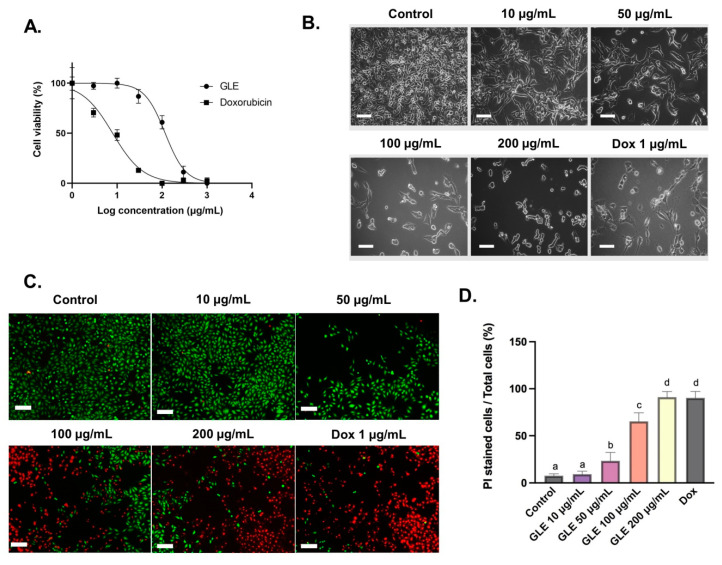
Cytotoxic effects of GLE in B16-F10 cells treated for 72 h: (**A**) The dose–response curve of GLE and doxorubicin (1–1000 µg/mL) cytotoxic activity in B16-F10 melanoma cells. (**B**) Morphological observation of B16-F10 melanoma cancer cells treated with GLE or doxorubicin for 72 h, scale = 50 µm. (**C**) Viability staining with calcein-AM (green) and PI (red) in B16-F10 murine melanoma cells treated with GLE or doxorubicin for 72 h. (**D**) The percentage of PI-stained cells represent dead cells. Different letters denote significant differences between treatments. Scale = 100 µm.

The cytotoxic effect of GLE on B16-F10 melanoma cells was evaluated with the MTT assay. GLE solution was diluted to various concentrations (1–1000 µg/mL), and effective doses were calculated from the dose–response curve in GraphPad Prism. After 72 h of administration, GLE shows moderate toxicity, with an IC_50_ value of 84.29 ± 1.93 µg/mL (Figure 3A). Based on US National Cancer Institute (NCI) guidelines, the value IC_50_ ≤ 20 µg/mL is considered highly cytotoxic, IC_50_ ranging between 21 and 200 µg/mL is considered moderately cytotoxic, IC_50_ ranging between 201 and 500 µg/mL is weakly cytotoxic, and IC_50_ above 500 µg/mL is considered to have no cytotoxic activity [50]. Hence, GLE could be considered moderately cytotoxic based on the presence of potential cytotoxic compounds. Furthermore, GLE treatment induces changes in the morphology of B16-F10 cells (Figure 3B). The cell death process is accompanied by changes in cell morphology, such as cell shrinkage and rounding. These features are observable in cells treated with GLE—especially at higher concentrations (50–200 µg/mL). In addition, our preliminary study showed the low cytotoxicity of GLE in NIH-3T3 normal fibroblast cells, with IC_50_ > 500 µg/mL (Figure S2). Previous cytotoxic analysis of extracts of the red macroalga *Gelidium amansii* showed growth-inhibitory effects at concentrations > 500 µg/mL after 7 days of treatment. No growth-inhibitory effect was seen at the concentration range of 500–7500 µg/mL after 3 days of treatment [51]. This actually indicates the low cytotoxic activity of *Gelidium* extracts in normal fibroblast cells. In addition, the low cytotoxic activity of macroalgae extracts and their phytochemical constituents in normal cells has been previously reported [52,53,54,55].

### 3.4. Cell Viability Analyses with Fluorescence Double Staining (Calcein-AM/PI)

Cell viability can be measured using the fluorescent probes calcein-AM and PI, which can differentiate between living and dead cells [9]. In living cells, intracellular esterase can convert calcein-AM to calcein, which stays in the living cells and emits green fluorescence. Meanwhile, PI is cell-impermeable—it can only penetrate cells with impaired plasma membrane integrity to bind with DNA and emit red fluorescence. The number of dead cells in B16-F10 cells treated with GLE was increased in a concentration-dependent manner. The semi-quantitative analyses were conducted with ImageJ software based on the images obtained via fluorescence-inverted microscopy (Figure 3C). The B16-F10 cells treated with higher doses of GLE (100–200 µg/mL) resulted in a significant reduction in the number of living cells and an increase in the number of dead cells (Figure 3D).

### 3.5. Effect of GLE on B16-F10 Apoptosis

**Figure 4:**
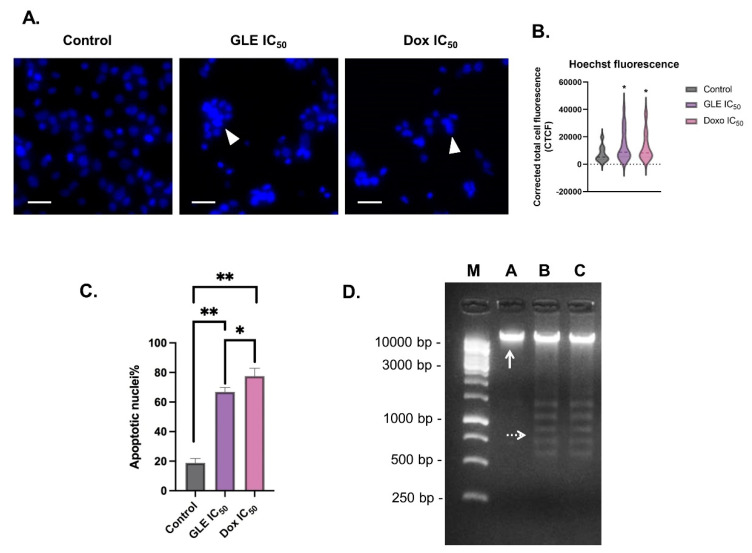
Apoptosis-inducing effects of GLE in B16-F10 melanoma cells: (**A**) Staining with Hoechst33342 reveals chromatin condensation in apoptotic cells; arrowheads = apoptotic nuclei. (**B**) Corrected total cell fluorescence (CTCF) values of B16-F10 cells treated with GLE and doxorubicin at IC_50_ concentrations. (**C**) Calculation of apoptotic nuclei percentage in B16-F10 cells treated with IC_50_ concentrations of GLE and doxorubicin. (**D**) Fragmentation of B16-F10 genomic DNA treated with GLE; a 1 kb DNA ladder (250–10,000 bp) was used as a standard; M: DNA 1 kb marker; A: control; B: GLE IC_50_ concentration; C: doxorubicin IC_50_ concentration. * Indicates significant difference compared to control (*p* < 0.05); ** indicates highly significant difference compared to control (*p* < 0.01). Arrowheads = apoptotic nuclei. White arrows indicate genomic DNA. White dotted arrows show fragmented DNA. Scale = 25 µm.

Chromatin condensation is one of the key features of cell apoptosis [56]. The fluorescence dye Hoechst33342 can be used to stain the condensed nuclei of apoptotic cells [57]. The B16-F10 cells treated with the IC_50_ concentration of GLE were seen to exhibit condensed nuclei (Figure 4A). To determine chromatin condensation between treatments, the fluorescence intensity of the treated cells could be calculated as corrected total cell fluorescence (CTCF) [39]. Based on the CTCF readout, there was a significant increase in fluorescence intensity in cells treated with GLE (CTCF = 13,676.31) compared to the control group (CTCF = 7390.22) (Figure 4B). Furthermore, the percentage of apoptotic cells treated with GLE (66.83 ± 3.06%) was higher than in the control group (18.83% ± 3.76%), but not significantly higher than in the doxorubicin-treated cells (77.50 ± 5.36%) (Figure 4C). In addition to nuclear DNA condensation, the fragmentation of DNA is also a key sign of cell apoptosis events, which can be observed via gel electrophoresis [40,58]. The formation of smaller DNA fragments can be observed in DNA samples of cells treated with IC_50_ concentrations of GLE and doxorubicin (Figure 4D).

### 3.6. Effects of GLE on Bak, Bax, and Bcl2 Expression

**Figure 5:**
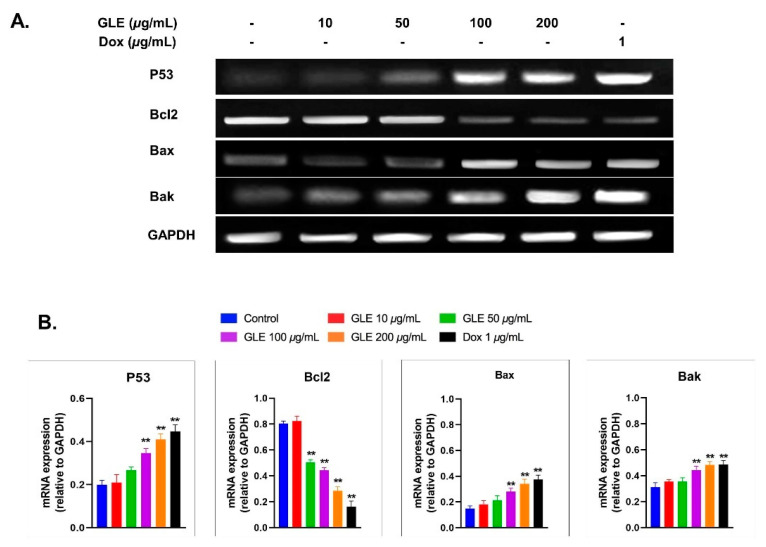
(**A**) Amplification of the apoptosis-related genes *p53*, *Bax*, *Bak*, and *Bcl2*. (**B**) Analyses of gene expression based on mRNA expression relative to the housekeeping gene GAPDH. ** indicates highly significant difference compared to control (*p* < 0.01).

The effect of GLE on the expression of apoptosis-related genes via RT-PCR was analyzed using Image Lab software (Figure 5A). GLE altered the expression of apoptosis-related genes after 72 h of treatment in B16-F10 melanoma cells. Compared to the untreated cells, pro-apoptotic mRNA levels were markedly increased. However, *Bcl2* expression was decreased by GLE treatment (Figure 5B). Several previous studies have shown that marine macroalgae extracts can alter the expression of apoptosis-related genes [59,60,61]. A previous study revealed the cytotoxic activity of various crude macroalgae extracts against MCF-10A [62]; among them are *Gelidium spinosum* and *Gelidium pulchellum*, which show very high cytotoxicity. However, to the best of our knowledge, no study has yet been conducted on the cytotoxic activity of *Gelidium latifolium* in melanoma cells.

## 4. Discussion

Macroalgae have been extensively investigated as sources of new bioactive chemicals with a variety of biological properties [63,64,65]. *Gelidium*, a red macroalgae genus, has previously been proven to inhibit cell proliferation in cultured cells [51,66,67]. *G. latifolium* is a red macroalga that is commonly found in the coastal areas of the island of Lombok, Indonesia; however, there remains limited information regarding its potential biological activity. In this study, we investigated the phytochemical properties and cytotoxic activity of *G. latifolium* ethanol extract (GLE) in B16-F10 melanoma cells.

Algal secondary metabolites such as phenolic compounds, polysaccharides, and polyunsaturated acids have been shown to have a wide variety of biological functions [9,53,68]. Due to the agar content of the red macroalga *Gelidium,* it is one of the most important edible marine algae [69]. *Gelidium* agar has also been proven in previous studies to have anti-obesity, antioxidant, and anticarcinogenic properties [24,70,71]. Thus, agar has been shown to possess a variety of biological activities with potential pharmacological and therapeutic applications. However, data on additional possible bioactive chemicals in *Gelidium* are quite limited. The red macroalga *G. latifolium* exhibits a high level of antioxidant activity when compared to other crude macroalgal preparations [72]. Some investigations have concluded that there is no association between free radical scavenging and cytotoxicity [73]; however, there are also other reports that show positive correlation between antioxidant and cytotoxic activity [74,75].

In our study, ESI-HR-TOF-MS analysis revealed a significant amount of brassicolene in the ethanol extract of native *Gelidium latifolium* from Lombok. Brassicolene is a diterpenoid found in the soft coral *Nephthea brassica* Kükenthal that has been shown to have cytotoxic effects in a variety of tumor cells [49,76]. Numerous studies have noted the presence of diterpenoids from marine macroalgae, and their cytotoxicity in various cancer cells [65,77,78]. However, this is the first study to demonstrate the presence of the diterpenoid brassicolene in the red alga *Gelidium latifolium*. Our preliminary results also show that GLE also contains putative fatty acids such as trans, cis-2,6-nonadienal, 12-oxo-trans-10-dodecenoic acid, palmitoleic acid, (2E)-2-tetradecenal, and eicosenoic acid (Figure S1). In addition, 2H-pyran and 5-methyl-2-octyl-3(2h)-furanone were also detected. All of the above were present at small levels compared to brassicolene. However, none of these compounds have been reported to exhibit antiproliferative activity in tumor cells. Hence, we suggest that brassicolene potentially contributes to GLE’s cytotoxic activity.

The main purpose of anticancer treatments is to kill the cancer cells without damaging normal cells. However, chemotherapy and radiotherapy possess limited efficacy, and exert their actions on both tumor and normal cells. This results in adverse side effects on patients, such as anemia, delirium, and peripheral neuropathy [79]. Thus, the development of a more effective treatment that has anticancer activity with lower cytotoxicity and fewer side effects is still needed. Natural products such as crude extracts from marine macroalgae are well studied for their moderate-to-low cytotoxic activity against various cancer cells [65].

Based on our results, GLE shows stronger cytotoxic activity compared to crude macroalgae extracts in other studies [53,80,81]. However, the cytotoxic activity of GLE was quite similar to *Laurencia papillosa*—125.8 ± 2.1 µg/mL and 121. 64 µg/mL, respectively [18]. The IC_50_ values could differ in different tumor models [82]; hence, in order to establish GLE antitumor activity, further investigations are needed in different cell lines. In addition, GLE shows low cytotoxic activity against normal murine fibroblast cells (Figure S2). Similar studies also showed the minimal cytotoxic activity of macroalgae extracts against non-tumorigenic cell lines [52,53,54,55]. The macroalga *Cystoseira tamariscifolia* also showed selective cytotoxic activity against the normal cell line HUVEC [82]. Our previous study also showed that the bioactive compound sulfated polysaccharide from red algae exerts no significant cytotoxic effect against HUVEC cells [9]; a possible reason for this is that the active substances of macroalgae interact with specific cancer-associated receptors or proteins, thus triggering a certain mechanism that causes cancer cell death [83]; however, further experiments are still needed in order to elucidate the precise mechanism.

Evading apoptosis is one of the hallmarks of cancer treatment to restrain the survival of abnormal cells. Hence, anticancer therapies commonly target apoptosis for the prevention and treatment of cancer. In general, the apoptotic pathway consists of several biochemical events, including the activation of apoptotic genes such as *p53*, *Bcl2*, *Bax*, and *Bak* [84]. Based on mRNA levels, GLE increased the expression of pro-apoptotic genes (i.e., *p53*, *Bax*, and *Bak*). Hence, GLE may promote apoptosis in cancer cells via a mitochondria-dependent intrinsic pathway. In addition, other essential regulatory proteins for apoptotic pathways include *Bcl2*. The mRNA expression of the anti-apoptotic gene *Bcl2* was decreased in B16-F10 cells treated with GLE. GLE concentrations above 100 µg/mL significantly increased the expression of *Bax* and *Bak*, while decreasing the expression of *Bcl2* (Figure 5B). This indicates that GLE potentially induces apoptosis via the mitochondrial apoptotic pathway [85].

## 5. Conclusions

Our study’s results show the potential pharmaceutical and medicinal value of the red macroalga *G. latifolium.* The ethanol extract of *G. latifolium* (GLE) shows promising phytochemical properties and antioxidant activity. GLE had a weaker antiproliferative activity compared to doxorubicin. The present study is based on the main component that is found in the *G. latifolium* ethanol extract—which is putatively brassicolene or a closely related molecule. Other minor bioactive compounds may interact with brassicolene in a synergistic or antagonistic manner. Hence, future research on the isolation of brassicolene would be welcome in order to better understand how it affects cell viability. Nevertheless, GLE cytotoxicity induced apoptosis based on morphological observation and altered expression of apoptosis-related genes. The upregulation of the pro-apoptotic gene *p53* and the downregulation of the anti-apoptotic gene *Bcl2* suggest that the mechanism of apoptosis takes place through an intrinsic pathway. At this point, our results show the presence of a potential bioactive compound that could be useful in the discovery of novel macroalgae-based antiproliferative compounds.

## Data Availability

The data presented in this study are available on request from the corresponding and first author.

## Supplementary Materials

The following are available online: Figure S1: HPLC analysis of GLE determined that brassicolene was substantially more present as a prominent peak than the other compounds; Figure S2: Cytotoxic effects of GLE in NIH-3T3 normal fibroblast cells treated for 72 h.

## Sample Availability

Samples of the compounds are available from the authors.
