# Supplementary material for: The Antiproliferative and Apoptosis-Inducing Effects of the Red Macroalgae Gelidium latifolium Extract against Melanoma Cells
Source: Molecules. 2021 Oct 30;26(21):6568. doi: 10.3390/molecules26216568 (PMC8587204; doi:10.3390/molecules26216568)
Supplement: Supplementary file 1 [file molecules-26-06568-s001.zip › molecules-1417135-supplementary.pdf]

Supplementary material.

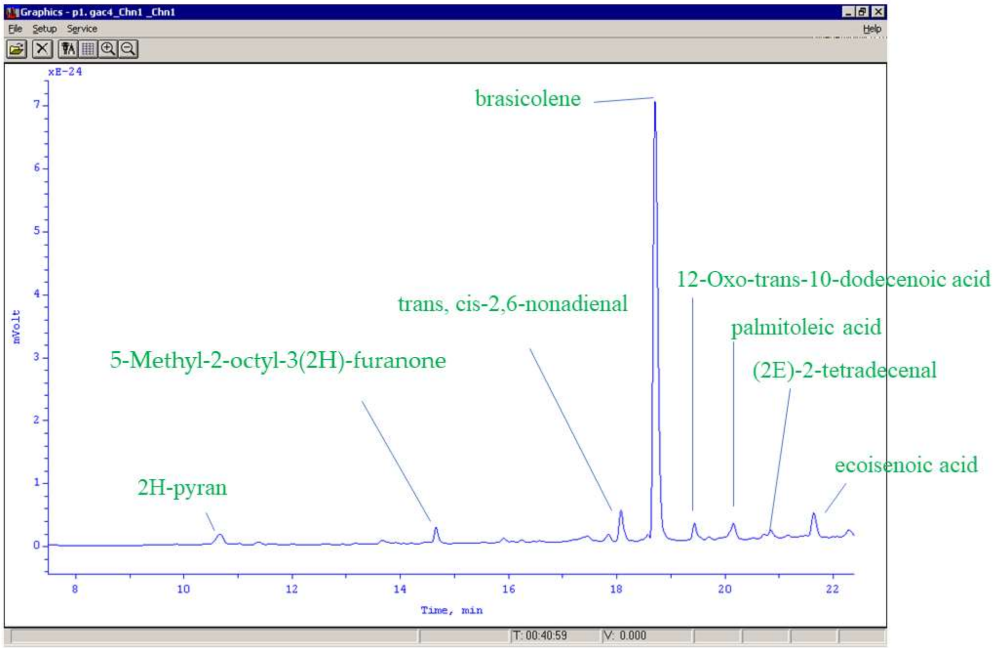

Figure S1. HPLC analysis on GLE determined that brassicolene was substantially more present as a prominent peak than the others. The majority of the substances were fatty acid putatives, trans, cis-2,6-nonadienal; 12-Oxo-trans-10-dodecenoic acid; palmitoleic acid, (2E)-2-tetradecenal, and ecoisenoic acid. In addition, 2H-pyran and furanone were also detected.

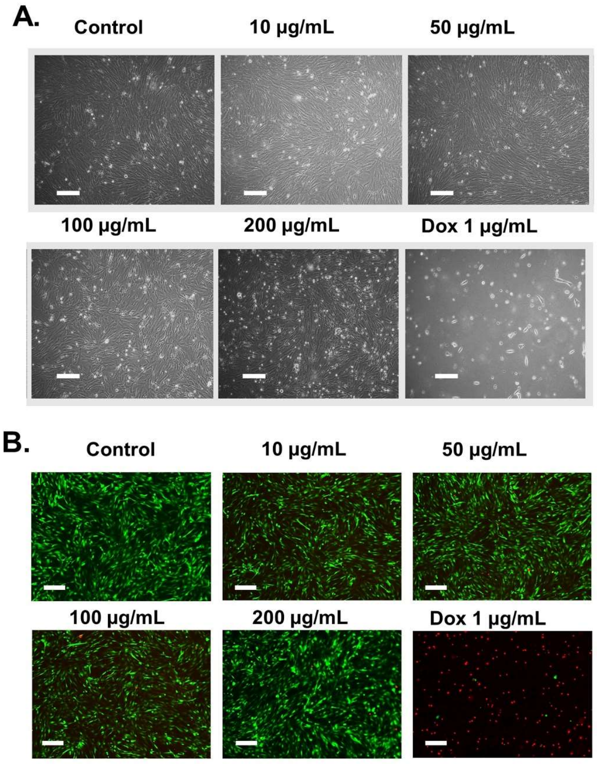

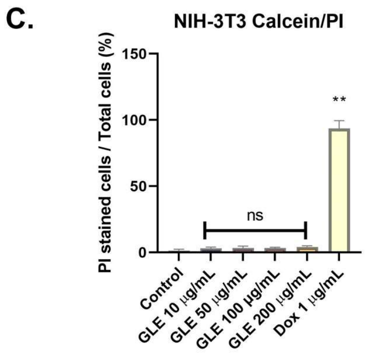

D.

Treatment	IC_{50}
GLE	>500 μg/mL
Doxorubicin	2.54 ± 0.67 μg/mL

Figure S2. Cytotoxic effects of GLE in NIH-3T3 normal fibroblast cells treated for 72 h. A. Morphological observation of NIH-3T3 fibroblast cells after GLE and doxorubicin for 72h, scale = 50 μm, B. Viability staining with Calcein-AM (green) and PI (red) in NIH-3T3 fibroblast cells treated with GLE or Doxorubicin for 72h, C. The percentage of PI-stained cells that are dead cells. D. Respective IC50 values of GLE and Doxorubicin against NIH-3T3 fibroblast cells. ** indicates significant difference (p<0.01) compared to control. ns indicates no significant difference compared to control group. Scale=100 μm.
